# Scientometric analyses of studies on the role of innate variation in athletic performance

**DOI:** 10.1186/2193-1801-3-307

**Published:** 2014-06-24

**Authors:** Michael P Lombardo, Shadie Emiah

**Affiliations:** Department of Biology, Grand Valley State University, Allendale, MI 49401 USA

**Keywords:** Scientometrics, Athletic performance, Innate variation, Genetic variation, Nature vs. nurture

## Abstract

Historical events have produced an ideologically charged atmosphere in the USA surrounding the potential influences of innate variation on athletic performance. We tested the hypothesis that scientific studies of the role of innate variation in athletic performance were less likely to have authors with USA addresses than addresses elsewhere because of this cultural milieu. Using scientometric data collected from 290 scientific papers published in peer-reviewed journals from 2000–2012, we compared the proportions of authors with USA addresses with those that listed addresses elsewhere that studied the relationships between athletic performance and (a) prenatal exposure to androgens, as indicated by the ratio between digits 2 and 4, and (b) the genotypes for angiotensin converting enzyme, α-actinin-3, and myostatin; traits often associated with athletic performance. Authors with USA addresses were disproportionately underrepresented on papers about the role of innate variation in athletic performance. We searched NIH and NSF databases for grant proposals solicited or funded from 2000–2012 to determine if the proportion of authors that listed USA addresses was associated with funding patterns. NIH did not solicit grant proposals designed to examine these factors in the context of athletic performance and neither NIH nor NSF funded grants designed to study these topics. We think the combined effects of a lack of government funding and the avoidance of studying controversial or non-fundable topics by USA based scientists are responsible for the observation that authors with USA addresses were underrepresented on scientific papers examining the relationships between athletic performance and innate variation.

## Background

Science advances best when scientists freely pursue questions of their own choosing. However, the subjects pursued by scientists are often influenced by their cultural milieu. For example, scientific progress is hindered when the questions pursued by scientists are explicitly or implicitly influenced by government regulations or societal norms (Loury 1994). Science journal editors can also influence the dissemination of knowledge by not publishing knowledge deemed either dangerous, prohibited by religious, moral or secular authorities, or obtained by means considered ethically unacceptable (i.e., “forbidden knowledge” (Journal editors et al. 2003; Kempner et al. 2005). For example, the controversy (Fauci and Collins 2012; Frankel 2012) that surrounded the publication of research demonstrating how to genetically alter in the laboratory the A/H5N1 strain of the influenza virus so that it is capable of airborne transmission in mammals thereby making it a potential biological weapon (Herfst et al. 2012) illustrates the complicated issues surrounding the publication of “forbidden knowledge”.

While government funding agencies, societal controls, and science journal editors may affect scientific inquiry into some questions, a more subtle form of the suppression of science is the self-censorship that results when scientists voluntarily avoid studying certain topics and publishing their data or ideas (Loury 1994). They may do this because they fear (a) violating social norms, (b) limiting their ability to obtain grants from government or private sources in the future, or (c) attracting the ire of their peers and the general public (Loury 1994; Hunter 2005; Kempner et al. 2005; Kempner 2008; Inbar and Lammers 2012). Peer anger and discrimination, both implicit and explicit, is a real phenomenon. In one study, social psychologists admitted they would discriminate against colleagues who held contrary ideological views during peer-review, hiring, and tenure decisions (Inbar and Lammers 2012).

Self-censorship is also a real phenomenon. Inbar and Lammers (2012) presented strong evidence of an inclination to self-censor by scientists studying controversial topics. Scientists from a variety of disciplines interviewed by Kempner et al. (2005) and Kempner (2008) admitted that they did not study controversial topics or disseminate knowledge that might provoke moral outrage, condemnation, or ostracism by their peers. Moreover, self-censorship was more prevalent than external constraints such as the legal prohibition of certain types of studies on scientific inquiry (Kempner et al. 2005). Self-censorship may be more injurious than external censorship to the advancement of science (Hunter 2005) because it is less transparent and more resistant to change because it typically occurs in response to powerful societal pressures (Loury 1994; Kempner et al. 2005; Kempner 2008). Ultimately, self-censorship by scientists results in the stagnation of science and the impoverishment of the public discourse of important issues (Loury 1994; Sowell 1995; Hunter 2005).

There is an ideologically charged atmosphere in the USA surrounding scientific explorations of the relationships between innate variation and athletic performance (Hoberman 1992, 1997; Entine 2000). The origins of this intellectual climate can be traced from a past history of slavery and its consequent explicit and implicit racism to a post-World War II academic and intellectual milieu that favored the hypothesis that variations in social and cultural environments during development are most responsible for individual differences in performance (i.e., the environmentalist paradigm) (Segerstråle 2000). Consequently, scientists and government funding agencies may avoid pursuing scientific questions about the potential role of innate variation in performance because of this intellectual and political environment. If a pervasive environmentalist sentiment in the USA has inhibited USA based investigators from pursing questions about the role of innate variation in athletic performance (i.e., self-censorship, *sensu* Loury (1994)), then we predict that USA based investigators will be underrepresented as authors on scientific papers about the role of innate variation in athletic performance compared to their representation on papers about the same factors independent of their role in athletic performance. Moreover, we predict that USA government funding agencies (e.g., National Institutes of Health (NIH), National Science Foundation (NSF)) will not solicit or fund grant proposals designed to directly pursue questions about the role of innate variation in performance but will solicit or grant proposals to study innate variation independent of performance. We tested these predictions two ways. First, we first compared the proportions of authors that listed USA addresses on papers published in peer-reviewed journals that examined the role of innate variation in athletic performance with the proportion of authors with USA addresses listed on papers that examined the same factors independent of their role in athletic performance. Second, we examined the patterns of solicitation of grant proposals and the funding for these topics by NIH and NSF.

Our study focused on four innate traits (a) prenatal exposure to testosterone as estimated by the digit ratio (i.e., the ratio between the lengths of digits 2 and 4) (DR) and the genotypes for the expression of the proteins (b) angiotensin-converting enzyme (ACE), (c) α-actinin-3 (ACTN3), and (d) myostatin (MSTN) that have often been associated with athletic performance (see below). Hereafter, the acronyms MSTN, ACE, and ACTN3 will be used to refer to both the genes and proteins for which they code. The evidence implicating the relationships between athletic performance and DR, ACE, ACTN3, and MSTN is summarized below.

### Digit ratio

The lengths of the second (index finger, 2D) and fourth (ring finger, 4D) digits are influenced by prenatal exposure to hormones. The length of 2D is positively influenced by prenatal exposure to estrogens whereas the length of 4D is positively influenced by exposure to testosterone (Manning et al. 2000; Manning 2002a). Consequently, the ratio between the lengths of these digits (i.e., 2D:4D) in human males is, on average, less than that of females across ethnic groups (Manning et al. 2000; Manning 2002a; Lutchmaya et al. 2004; Manning et al. 2007a). Athletic prowess in both males and females, as indicated by performance in tests of physical skills (Fink et al. 2006; Hönekopp et al. 2007; Manning et al. 2007b; Manning and Hill 2009) and level of athletic achievement (Manning 2002b; Pokrywka et al. 2005; Tester and Campbel 2007), is negatively correlated with 2D:4D (Manning and Taylor 2001; Hönekopp et al. 2006; Hönekopp and Schuster 2010).

### Angiotensin converting enzyme (ACE)

Angiotensin converting enzyme helps regulate circulatory homeostasis through the synthesis of vasoconstrictor angiotensin II and the breakdown of vasodilator kinins (Myerson et al. 1999) and is a widely studied in the context of athletic endurance performance. The ACE gene has two alleles, I and D. The I allele is associated with lower serum and tissue ACE activity resulting in improved fatigue resistance during exercise (Myerson et al. 1999). The D allele is associated with greater ACE activity, increased muscle volume and strength, greater training-related increases in muscle volume and strength than the I allele (Myerson et al. 1999; Ahmetov and Rogozkin 2009; Lippi et al. 2009). These patterns suggest that in some populations the presence of the I allele predisposes some individuals for success in endurance events whereas the D allele predisposes others for success in sprinting and other power-dependent athletic events (Myerson et al. 1999). A recent meta-analysis demonstrated an association between the ACE homozygous II genotype and performance in endurance athletic events (Ma et al. 2013).

### α-actinin-3 (ACTN3)

α-actinin-3 is an actin binding protein whose presence is restricted to sarcomeric Z-line of fast-twitch (Type II) skeletal muscle fibers stabilizing these fibers during contraction resulting in faster, stronger contractions (Mills et al. 2001). Two alleles, R and X, have been discovered at the ACTN3 locus. An association between *ACTN3* genotype and sprint performance has been demonstrated in numerous studies. Relative to non-athlete and non-elite athlete controls, the homozygous RR genotype, with few exceptions (Lucia et al. 2007; Druzhevskaya et al. 2008), is more common in elite sprint/power athletes (Yang et al. 2003; MacArthur and North 2004; Niemi and Majamaa 2005) whereas the homozygous XX genotype is associated with elite athlete endurance performance in some populations (Yang et al. 2003; Eynon et al. 2009). A recent meta-analysis demonstrated an association between the presence of the ACTN3 R allele and performance in sprint/power athletic events (Ma et al. 2013).

### Myostatin (MSTN)

The MSTN gene, a member of the transforming growth factor-β super family of secreted growth and differentiation factors located on chromosome 2 in humans, is highly conserved in vertebrates and inhibits excessive skeletal muscle growth (McPherron et al. 1997; Lee and McPherron 1999, 2001; McNally 2004; Girgenrath et al. 2005; Rodgers and Garikipati 2008). Athletic performance in animals is associated with MSTN. For example, whippets (Mosher et al. 2007) and thoroughbred horses (Hill et al. 2010) lacking MSTN display superior sprint performance.

The relationship between MSTN genotype and athletic performance in humans is less clear (Lee 2007). However, the following observations suggest that MSTN expression may affect athletic performance: (a) Resistance training results in varying degrees of decreased MSTN expression (Walker et al. 2004; Jespersen et al. 2009). (b) MSTN genotypes were associated with baseline muscle strength and size in a small sample of African-Americans (Kostek et al. 2009). (c) MSTN genotype was associated with the ability to produce “peak” power during muscle contractions during stationary jumping in a population of young, non-athletic Caucasian men in Spain (Santiago et al. 2011). In contrast, the null MSTN mutation that would result in exaggerated musculature was not found in a survey of elite and high-level body builders in the USA (Liu et al. 2005).

Although scientific support for an association between human athletic performance and MSTN deficiency or genotype remains limited, athletes and coaches are very interested in the effects of the genotype on performance. A Google search using the keywords “myostatin” and “strength” returned ~ 108,000 results on 30 April 2014. Because MSTN inhibits muscle growth, athlete anti-doping agencies (e.g., World Anti-Doping Agency, http://www.wada-ama.org) are concerned that athletes and their coaches may attempt to enhance performances in strength and power dependent athletic events (e.g., combat sports, jumping, sprinting, throwing, weightlifting,) by using pharmaceutical MSTN inhibitors or via “gene doping” techniques (Fedoruk and Rupert 2008). The WADA list of prohibited substances includes agents that modify or inhibit MSTN function (http://www.wada-ama.org).

## Results

### Athletic performance and DR

Twenty-two of the 33 (67%) papers located by the Web of Science met our inclusion criteria for papers about the relationship between athletic performance and DR (Table 1). These papers appeared in 15 different journals (1.50 ± 0.89 papers per journal) and had 56 different authors (3.05 ± 1.29 authors per paper) from 12 different countries (1.27 ± 0.55 countries represented per paper and 4.67 ± 4.23 authors per country) (Table 1). We used a random number generator to select 22 of the 280 (8%) papers about DR independent of athletic performance found by the Web of Science (Table 1). These papers appeared in 16 different journals (1.38 ± 0.62 papers per journal) and had 74 different authors (3.91 ± 1.74 authors per paper) from 12 different countries (6.62 ± 6.08 authors per country) (Table 1).

**Table 1:** **Summary of the results of ISI Web of Science searches for papers published between 1 January 2000 and 31 December 2012 using keywords “athlete(s)”, “athletic performance”, “sport(s)”, “digit ratio”, “myostatin”, “MSTN”, “angiotensin converting enzyme”, “ACE”, “α-actinin-3”, and “ACTN3”**

	Papers found using search criteria	Papers meeting inclusion criteria			
Search	N	N	Number of different journals	Number of different authors	Number of countries that authors listed as addresses
Athletic performance and					
Digit ratio	33	22	15	56	12
ACE	141	74	43	400	29
ACTN3	94	49	30	222	26
MSTN	39	15	10	89	12
Total	307	160			
Without athletic performance					
Digit ratio	280	22	16	74	12
ACE	22,031	74	64	435	30
ACTN3	38	19	17	122	10
MSTN	1261	15	14	89	9
Total	23,610	130			

See text for search and inclusion criteria.

Although the differences between the proportions were large, first authors and corresponding authors with USA addresses were as likely to be found on papers about athletic performance and DR as on those about DR independent of athletic performance (Table 2). However, the achieved statistical power of these tests was low (1 – β = 0.11 and 0.22, respectively). In contrast, when all authors of all papers about DR were considered, authors that listed USA addresses were found significantly less often on papers about athletic performance and DR than were found on papers about DR independent of athletic performance (Table 2).

**Table 2:** **National addresses of authors listed on papers published between 1 January 2000 and 31 December 2012 about digit ratio (DR), and angiotensin converting enzyme (ACE), α-actinin-3 (ACTN3) and myostatin (MSTN) genotypes that were either in the context of athletic performance or independent of athletic performance**

Innate factor	Category of author	Was the paper about the relationship between the innate factor and athletic performance?	Authors with USA addresses (%)	Authors with non-USA addresses	Total	Fisher exact test
DR	First author	Yes	3 (13.6%)	19	22	P = 0.46
		No	6 (27.3%)	16	22	
	Corresponding author	Yes	3 (13.6%)	19	22	P = 0.28
		No	7 (31.8%)	15	22	
	All authors	Yes	7 (12.5%)	49	56	P = 0.03
		No	21 (28.4%)	53	74	
ACE	First author	Yes	4 (5.3%)	70	74	P < 0.0001
		No	25 (33.8%)	49	74	
	Corresponding author	Yes	4 (5.3%)	70	74	P < 0.0001
		No	25 (33.8%)	49	74	
	All authors	Yes	28 (7.0%)	372	400	P < 0.0001
		No	117 (26.9%)	318	435	
ACTN3	First author	Yes	1 (2.0%)	48	49	P = 0.006
		No	5 (26.3%)	14	19	
	Corresponding author	Yes	1 (2.0%)	48	49	P = 0.006
		No	5 (26.3%)	14	19	
	All authors	Yes	18 (8.1%)	204	222	P < 0.0001
		No	36 (29.5%)	86	122	
Myostatin	First author	Yes	2 (13.3%)	13	15	P = 0.22
		No	6 (40.0%)	9	15	
	Corresponding author	Yes	2 (13.3%)	13	15	P = 0.11
		No	7 (46.7%)	8	15	
	All authors	Yes	18 (20.2%)	71	89	P = 0.005
		No	36 (40.4%)	53	89	
All papers pooled	First author	Yes	10 (6.3%)	150	160	P < 0.0001
		No	42 (32.3%)	88	130	
	Corresponding author	Yes	10 (6.3%)	150	160	P < 0.0001
		No	44 (33.8%)	86	130	
	All authors	Yes	60 (9.6%)	565	625	P < 0.0001
		No	209 (28.8%)	516	725	

Fisher exact tests tested the null hypotheses that whether first, corresponding, or all authors had either USA or non-USA addresses was independent of whether a paper’s focus was on the relationship between an innate factor and athletic performance or an innate factor independent of its relationship to athletic performance.

The largest number of authors on papers about the relationship between athletic performance and DR listed the United Kingdom as their address (Table 3). The proportion of authors that listed United Kingdom addresses on papers about the relationship between athletic performance and DR did not significantly differ from the proportion of authors that listed non-United Kingdom addresses on papers about DR independent of athletic performance (Table 3). The achieved statistical power of this test was low (1 – β = 0.137). Similarly, the largest number of authors on papers about DR independent of athletic performance listed the United Kingdom as their address (Table 4). Again, the proportion of authors that listed United Kingdom addresses on papers about the relationship between athletic performance and DR did not significantly differ from the proportion of authors that listed non-United Kingdom addresses on papers about DR independent of athletic performance (Table 4). The achieved statistical power of this test was low (1 – β = 0.137).

**Table 3:** **National addresses of authors listed on papers published between 1 January 2000 and 31 December 2012 about digit ratio (DR), and angiotensin converting enzyme (ACE), α-actinin-3 (ACTN3) and myostatin (MSTN) genotypes that were either in the context of athletic performance or independent of athletic performance**

	Country			
Innate factor	Authors with United Kingdom address (%)	Authors with non-United Kingdom address	Total	Fisher exact test
DR and athletic performance	17 (30.4%)	39	56	P = 0.42
DR independent of athletic performance	17 (22.9%)	57	74	
	Authors with Spain address (%)	Authors with non-Spain address		
ACE and athletic performance	58 (14.5%)	342	400	P < 0.0001
ACE independent of athletic performance	1 (0.2%)	434	435	
	Authors with Italy address (%)	Authors with non-Italy address		
ACE and athletic performance	58 (14.5%)	342	400	P < 0.0001
ACE independent of athletic performance	22 (5.1%)	413	435	
	Authors with Spain address (%)	Authors with non-Spain address		
ACTN3 and athletic performance	36 (16.2%)	186	222	P = 0.64
ACTN3 independent of athletic performance	17 (13.9%)	105	122	
	Authors with Germany address (%)	Authors with non-Germany address		
MSTN and athletic performance	17 (19.1%)	72	89	P < 0.0001
MSTN independent of athletic performance	0 (0%)	89	89	
All papers pooled	Authors with Spain address (%)	Authors with non-Spain address		
All factors and athletic performance	74 (11.7%)	561	635	P < 0.0001
All factors independent of athletic performance	18 (2.5%)	707	725	

Country indicates for each innate factor the country, other than the USA, that authors most frequently listed as their address on papers about the relationship between athletic performance and each innate factor. For all papers pooled, country indicates the country, other than the USA, that authors most frequently listed as their address on papers about the relationship between athletic performance and the innate factors pooled together. Fisher exact tests tested the null hypotheses that whether authors listed had either “country” or “non-country” addresses was independent of whether a paper’s focus was about the relationship between an innate factor and athletic performance or an innate factor independent of its relationship to athletic performance.

**Table 4:** **National addresses of authors listed on papers published between 1 January 2000 and 31 December 2012 about digit ratio (DR), and angiotensin converting enzyme (ACE), α-actinin-3 (ACTN3) and myostatin (MSTN) genotypes that were either in the context of athletic performance or independent of athletic performance**

	Country			
Innate factor	Authors with United Kingdom address (%)	Authors with non-United Kingdom address	Total	Fisher exact test
DR and athletic performance	17 (30.4%)	39	56	P = 0.42
DR independent of athletic performance	17 (22.9%)	57	74	
	Authors with Japan address (%)	Authors with non-Japan address		
ACE and athletic performance	29 (7.3%)	371	400	P = 0.072
ACE independent of athletic performance	48 (11.0%)	387	435	
	Authors with Spain address (%)	Authors with non-Spain address		
ACTN3 and athletic performance	36 (16.2%)	186	222	P = 0.64
ACTN3 independent of athletic performance	17 (13.9%)	105	122	
	Authors with Norway address (%)	Authors with non-Norway address		
MSTN and athletic performance	0 (0%)	89	89	P < 0.0015
MSTN independent of athletic performance	10 (11.2%)	79	89	
	Authors with Japan address (%)	Authors with non-Japan address		
All factors pooled and athletic performance	38 (5.9%)	597	635	P = 0.0071
All factors independent of athletic performance	73 (10.1%)	652	725	

Country indicates for each innate factor the country, other than the USA, that authors most frequently listed as their address on papers about each innate factor independent of athletic performance. For all papers pooled, country indicates the country, other than the USA, that authors most frequently listed as their address on papers about the innate factors independent of athletic performance. Fisher exact tests tested the null hypotheses whether authors listed had either “country” or “non-country” addresses was independent of whether a paper’s focus was about the relationship between an innate factor and athletic performance or an innate factor independent of its relationship to athletic performance.

The proportion of authors with United Kingdom addresses on papers about the relationship between DR and athletic performance was twice that of authors with USA addresses but because of small sample sizes this difference was not statistically significant (Tables 5 and 6). The achieved power of these statistical tests was low (1 – β = 0.473).

**Table 5:** **National addresses of authors listed on papers published between 1 January 2000 and 31 December 2012 about digit ratio (DR), and angiotensin converting enzyme (ACE), α-actinin-3 (ACTN3) and myostatin (MSTN) genotypes that were either in the context of athletic performance or independent of athletic performance**

	Innate factor			
Country	DR and athletic performance (%)	DR independent of athletic performance	Total	Fisher exact test
USA	7 (25.0%)	21	28	P = 0.07
United Kingdom	17 (50.0%)	17	34	
	ACE and athletic performance (%)	ACE independent of athletic performance		
USA	28 (19.3%)	117	145	P < 0.0001
Spain	58 (98.3%)	1	59	
USA	28 (19.3%)	117	145	P < 0.0001
Italy	58 (72.5%)	22	80	
	ACTN3 and athletic performance (%)	ACTN3 independent of athletic performance		
USA	18 (33.3%)	36	54	P = 0.0005
Spain	36 (67.9%)	17	53	
	MSTN and athletic performance (%)	MSTN independent of athletic performance		
USA	18 (33.3%)	36	89	P < 0.0015
Germany	10 (11.2%)	79	89	
	All factors and athletic performance (%)	All factors independent of athletic performance		
USA	60 (28.7%)	209	269	P < 0.0001
Spain	74 (80.4%)	18	92	

Country indicates for each innate factor the country, other than the USA, that authors most frequently listed as their address on papers about the relationship between athletic performance and each innate factor. For all papers pooled, country indicates the country, other than the USA, that authors most frequently listed as their address on papers about the relationship between athletic performance and each innate factor. Fisher exact tests tested the null hypotheses whether authors listed had either USA or other “country” addresses was independent of whether a paper’s focus was about the relationship between an innate factor and athletic performance or an innate factor independent of its relationship to athletic performance.

**Table 6:** **National addresses of authors listed on papers published between 1 January 2000 and 31 December 2012 about digit ratio (DR), and angiotensin converting enzyme (ACE), α-actinin-3 (ACTN3) and myostatin (MSTN) genotypes that were either in the context of athletic performance or independent of athletic performance**

	Innate factor			
Country	DR and athletic performance (%)	DR independent of athletic performance	Total	Fisher exact test
USA	7 (25.0%)	21	28	P = 0.07
United Kingdom	17 (50.0%)	17	34	
	ACE and athletic performance (%)	ACE independent of athletic performance		
USA	28 (19.3%)	117	145	P < 0.0001
Japan	29 (37.6%)	48	49	
	ACTN3 and athletic performance (%)	ACTN3 independent of athletic performance		
USA	18 (33.3%)	36	54	P = 0.0005
Spain	36 (67.9%)	17	53	
	MSTN and athletic performance (%)	MSTN independent of athletic performance		
USA	18 (33.3%)	36	89	P = 0.0504
Norway	0 (0%)	10	10	
	All factors and athletic performance (%)	All factors independent of athletic performance		
USA	60 (28.7%)	209	269	P < 0.0001
Japan	73 (65.8%)	38	111	

Country indicates for each innate factor the country, other than the USA, that authors most frequently listed as their address on papers about each innate factor independent of athletic performance. For all papers pooled, country indicates the country, other than the USA, that authors most frequently listed as their address on papers about the innate factors independent of athletic performance. Fisher exact tests tested the null hypotheses whether authors listed had either USA” or “other country” addresses was independent of whether a paper’s focus was about the relationship between an innate factor and athletic performance or an innate factor independent of its relationship to athletic performance.

### Athletic performance and ACE

Seventy-four of the 141 (52%) papers located by the Web of Science met our inclusion criteria for papers about the relationship between athletic performance and ACE (Table 1). These papers appeared in 43 different journals (1.72 ± 1.79 papers per journal) and had 400 different authors (7.22 ± 2.60 authors per paper) from 29 different countries (1.50 ± 0.80 countries represented per paper and 13.79 ± 16.63 authors per country) (Table 1). We used a random number generator to select 74 of the 22,031 (0.03%) papers about ACE independent of athletic performance found by the Web of Science (Table 1). These papers appeared in 64 different journals (1.10 ± 0.46 papers per journal) and had 435 different authors (5.88 ± 3.26 authors per paper) from 30 different countries (14.60 ± 22.57 authors per country) (Table 1).

When all ACE papers were considered, the proportions of first authors, corresponding authors, and all authors with USA addresses were significantly smaller on papers about athletic performance and ACE than on papers about ACE independent of athletic performance (Table 2).

Authors with addresses in Spain and Italy represented the largest numbers of authors on papers about the relationship between athletic performance and ACE; authors from each country represented 14.5 percent (29 percent total) of the 400 authors listed (Table 3). Compared to authors with addresses elsewhere, authors with addresses in Spain or Italy were disproportionately underrepresented among authors listed on papers about ACE independent of athletic performance (Table 3). However, compared to authors with USA addresses, authors with addresses in Spain or Italy were significantly more likely to be found on papers about the relationship between ACE and athletic performance (Table 5).

Authors with Japanese addresses made-up the largest number of authors listed on papers about the ACE independent of athletic performance (Table 4). However, they were as equally likely to be listed on papers about the relationship between ACE and athletic performance as were authors that listed addresses elsewhere (Table 4). The achieved power of this statistical test was low (1 – β = 0.431). However, compared to authors with USA addresses, a significantly larger proportion of authors with Japanese addresses were listed on papers about the relationship between ACE and athletic performance (Table 6).

### Athletic performance and ACTN3

Forty-nine of the 94 (52%) papers located by the Web of Science met our inclusion criteria for papers about the relationship between athletic performance and ACTN3 (Table 1). These papers appeared in 30 different journals (1.63 ± 1.38 papers per journal) and had 222 different authors (6.86 ± 2.91 authors per paper) from 26 different countries (1.76 ± 1.03 countries represented per paper and 8.53 ± 7.95 authors per country) (Table 1). We collected scientometric data from 19 of the 38 (50%) papers found by the Web of Science that were about ACTN3 independent of athletic performance (Table 1) and met our inclusion criteria (see Methods). These papers appeared in 17 different journals (1.12 ± 0.33 papers per journal) and had 122 different authors (7.11 ± 2.08 authors per paper) from 10 different countries (12.10 ± 9.67 authors per country) (Table 1).

When all ACTN3 papers were considered, the proportions of first authors, corresponding authors, and all authors with USA addresses were significantly smaller on papers about athletic performance and ACE than were those found on papers about ACE independent of athletic performance (Table 2).

Authors with addresses in Spain made-up the largest number of authors listed on papers about the relationship between athletic performance and ACTN3 (Table 3). Authors with Spanish addresses were equally likely to be listed on papers about the relationship between ACTN3 and athletic performance as they were on papers about ACTN3 independent of athletic performance (Tables 3, 4). The achieved statistical power of these tests was low (1 – β = 0.069). However, compared to authors with USA addresses, authors with addresses in Spain were significantly more likely to be found on papers about the relationship between ACTN3 and athletic performance (Tables 5, 6).

### Athletic performance and MSTN

Fifteen of the 39 (38%) papers located by the Web of Science met our inclusion criteria for papers about the relationship between athletic performance and MSTN (Table 1). These papers appeared in 10 different journals (1.50 ± 0.97 papers per journal) and had 89 different authors (6.20 ± 3.61 authors per paper) from 12 different countries (1.53 ± 1.06 countries represented per paper and 7.42 ± 6.32 authors per country) (Table 1). We used a random number generator to select 15 of the 1261 (1%) papers found by the Web of Science that were about MSTN independent of athletic performance (Table 1). These papers appeared in 14 different journals (1.08 ± 0.28 papers per journal) and had 89 different authors (6.27 ± 2.43 authors per paper) from 9 different countries (9.89 ± 10.11 authors per country) (Table 1).

Although the differences between the proportions were large, first authors and corresponding authors were as likely to list USA as non-USA addresses (Table 2). However, the achieved statistical power of these tests was low (1 – β = 0.23 and 0.36, respectively; Table 2). In contrast, when all MSTN papers were pooled together, authors with USA addresses were found significantly less often on papers about athletic performance and MSTN than they were on papers about MSTN independent of athletic performance (Table 2).

Authors with addresses in Germany represented the largest proportion of the authors listed on papers about the relationship between athletic performance and MSTN (Table 3). No authors with German addresses were listed on papers about MSTN independent of athletic performance (Table 3). In contrast, authors that listed their address as Germany were found significantly less often than were authors with USA addresses on papers about the relationship between MSTN and athletic performance (Table 5).

The largest proportion of authors on papers about MSTN independent of athletic performance listed Norway as their address (Table 4). No authors with addresses in Norway were listed on papers about the relationship between MSTN and athletic performance (Table 4) and authors with Norwegian addresses were found significantly less often than those with non-Norwegian addresses on papers about the relationship between MSTN and athletic performance (Table 4). However, authors with addresses in Norway were as likely as authors with USA addresses to be found on papers about the relationship between MSTN and athletic performance (Table 6). The achieved statistical power of this comparison was low (1 – β = 0.596).

### Athletic performance and all innate factors pooled together

When all papers were pooled together for analyses (n = 160; Table 1), first, corresponding, and all listed authors with USA addresses were found significantly less often on papers about the relationship between athletic performance and innate factors than they were found on papers about innate factors independent of athletic performance (Table 2).

The greatest proportion of authors on the combined sample of papers that examined the relationship between athletic performance and the four innate factors listed addresses in Spain (Table 3). Authors with Spanish addresses were significantly more likely to be found on papers that examined the relationship between athletic performance and the four innate factors pooled together than were authors (a) with addresses from elsewhere (Table 3) or (b) with USA addresses (Table 5).

The greatest proportion of authors on the pooled sample of papers that examined the four innate factors independent of athletic performance listed addresses in Japan (Table 4). Authors with Japanese addresses were found significantly less often on papers that examined the relationship between athletic performance and the four innate factors combined than were authors with addresses elsewhere (Table 4). However, authors with Japanese addresses were more likely than authors with USA addresses to be found on papers about the relationship between athletic performance and the innate factors (Table 6).

### USA government funding of research on the relationships between athletic performance and DR, ACE, ACTN3, or MSTN

Between 2000–2012, neither NIH nor NSF funded any grant proposals designed to directly examine the relationships between athletic performance and DR, ACE, ACTN3, or MSTN. During the same time period, (a) NIH funded projects examining the biomedical correlates of DR (n = 1), ACE (n = 416), ACTN3 (n = 3), and MSTN (n = 84) and NSF funded 3 projects that proposed to examine MSTN in nonhuman animals and (b) NIH funded 152 grant proposals designed to examine hypertension and race, 280 grant proposals designed to examine type 2 diabetes and race, and another 114 grant proposals that had the term “racial differences” in their titles or abstracts. No grants were funded that had the following combination of terms in their titles or abstracts: “athlete and race”, “athlete and racial differences”, “athletic performance and race”, and “athletic performance and racial differences”.

Between 2000–2012, NIH did not publish any Program Announcements (PA), Request for Application (RFA), or Request for Proposal (RPP) notices that solicited applications or proposals designed to directly examine the relationships between athletic performance and DR, ACE, ACTN3, or MSTN. A PA is a formal statement from NIH about a new or ongoing extramural activity or program, an RFA is a formal statement from NIH that solicits grants or cooperative agreement applications in a well-defined scientific area, and an RPP is an announcement from NIH that it wishes to award a contract to meet a specific need (http://www.grants.nih.gov/glossary). Thus, NIH did not appear to be adverse to funding studies related to sports medicine between 2000–2012 and published 3 PAs and 1 RFA soliciting proposals to study phenomena related to sports medicine, including women’s health in sports and exercise (PA-02-115) and protein interactions governing membrane transport in pulmonary health and disease (RFA-DK-01-012, PA-06-076, PA-07-137). The PA most relevant to our study, PA-02-115, did not mention studying the possibility that innate factors could influence sports prowess.

## Discussion

### Scientists with USA addresses were underrepresented on papers about the relationships between athletic performance and innate variation

The scientometric data presented here demonstrate that compared to scientists with addresses elsewhere, scientists with USA addresses were disproportionately underrepresented as first authors, corresponding authors, and other authors listed on scientific papers about the relationships between athletic performance and innate variation in DR, ACE, ACTN3, or MSTN. These patterns of authorship were different from those of authors with addresses in other countries that were most frequently listed on papers on these topics (e.g. Germany, Italy, Japan, Norway, Spain, and the United Kingdom). Generally, authors with addresses in these countries (a) were either equally likely to be found or disproportionately more often found on papers that examined the relationship between the four innate factors and athletic performance compared to papers about the innate factors independent of athletic performance and (b) had a significantly greater proportional representation than did authors with USA addresses on papers about the relationship of the innate factors and athletic performance. However, there were several exceptions to this pattern.

First, authors with addresses in Norway were never listed on papers about the relationship between MSTN and athletic performance (Table 4). Consequently, they were disproportionately found listed on papers about MSTN independent of athletic performance (Table 4). However, the sample size of authors with addresses in Norway was small (n = 10). Second, authors with addresses in the United Kingdom were equally likely as those with addresses in the USA to be found on papers about the relationship between DR and athletic performance (Table 6). However, probably because of small sample sizes, the difference was not statistically significant (Table 6). Third, authors that listed addresses in Germany were never found on papers about MSTN independent of athletic performance (Table 3). As a consequence, authors with addresses in Germany were less likely than authors with USA addresses to be found on papers about the relationship between MSTN and athletic performance (Table 5). This was the only instance of authors with USA addresses having a significantly greater proportional representation on papers about the relationship between an innate factor and athletic performance. Last, authors with Japanese addresses were about twice as likely to be found on papers about the innate factors independent of athletic performance than on papers about the relationship between the innate factors and athletic performance (Table 4). Nevertheless, authors with Japanese addresses were more likely than those with USA addresses to be found on the pooled sample of papers that examined the relationship between the innate factors and athletic performance (Table 6). Taken together, these patterns suggest that authors based in these countries were more likely than authors with USA addresses to publish papers that examined questions about the roles of these innate factors in athletic performance.

### Why are USA based scientists underrepresented as authors of papers on the relationships between athletic performance and DR, ACE, ACTN3, or MSTN?

Why didn’t scientists from USA based laboratories publish a comparable proportion of papers on the relationships between athletic performance and DR, ACE, ACTN3, or MSTN? We consider several possible explanations for this pattern.

First, perhaps the disproportionately small representation of papers from USA based authors about the relationships between athletic performance and DR, ACE, ACTN3, or MSTN because there are relatively few sports scientists in the USA? This is not a viable explanation. There are tens of thousands of American scientists interested in sports sciences and sports medicine; 90% of the 45,000 members of the American College of Sports Medicine reside in the USA (http://www.acsm.org).

Second, perhaps scientists working at USA based laboratories were simply not interested in sports so were not motivated to scientifically pursue questions about the relationships between athletic performance and innate variation in DR, ACE, ACTN3, or MSTN? This explanation is unlikely because, in general, sports interest is high in the USA. For example, Americans (a) participate in sports in large numbers (http://www.census.gov/hhes/school/data/cps/2010/tables.html; http://www.ayso.org/AboutAYSO/history.aspx; http://www.littleleague.org/learn/about/historyandmission/aroundtheworld.htm; http://www.ncaapublications.com/p-4334-1981-82-2012-13-ncaa-sports-sponsorship-and-participation-rates-report.aspx; Deaner et al. 2012), (b) are avid avid sports fans (pewresearch.org; http://www.census.gov/compendia/statab/cats/arts_recreation_travel/recreation_and_leisure_activities; http://nielsen.com/us/en/newswire/2011/ and (c) spend large sums of money on sporting equipment and activities (http://www.census.gov/compendia/statab/cats/arts_recreation_travel/recreation_and_leisure_activities.). Collectively, these data demonstrate that the citizens of the USA devote considerable time, energy, and resources on sports activities. Even if USA scientists are less interested in sports than other Americans, we do not think that the lack of published research originating from USA based laboratories on the relationships between athletic performance and DR, ACE, ACTN3, or MSTN is primarily due to a lack of interest in sport sciences by USA scientists.

Third, a lack of government funding could be a proximate explanation for the lack of USA based published research examining the relationships between athletic performance and innate variation in DR, ACE, ACTN3, or MSTN. This is a viable, and important, explanation; neither NIH nor NSF reported funding research on these topics during the study period and NIH did not solicit any proposals to study these topics. Neither NIH nor NSF explicitly restricts funding from projects that propose to examine the relationships between athletic performance and innate variation. However, the project titles and abstracts of grant proposal not funded by NIH or NSF needed to test the hypothesis that cryptic funding restrictions (i.e., external censorship) are partly responsible for the disproportionately small number of papers originating from USA based laboratories are not easily available.

A lack of funding would negatively influence the pursuit of research on the relationship between athletic performance and innate factors for at least two reasons. First, contemporary scientific research is relatively expensive inhibiting scientists interested in these topics from pursuing them without financial support. Second, hiring, tenure, and promotion decisions at many academic institutions with a research mission in the USA are influenced, at least in part, by the ability of scientists to obtain funding from NIH or NSF and publish their results in peer-reviewed scientific journals thereby further inhibiting them from pursuing this kind of research.

It is not obvious why NIH and NSF did not fund research about the relationships between athletic performance and DR, ACE, ACTN3, or MSTN during the sample period. One possible explanation is that no researchers submitted grant proposals requesting funding to examine these topics. This hypothesis cannot be evaluated, but NIH did not solicit any proposals on these topics. Descriptions of the types of research funded by these agencies does not automatically preclude them from providing funding. The NIH, an agency of the US Department of Health and Human Services, is primarily responsible for biomedical and health-related research, with the goal of acquiring new knowledge to help prevent, detect, diagnose, and treat disease and disability (http://www.nih.gov). Although examining the biomedical correlates of DR, ACE, ACTN3, or MSTN in healthy individuals, including athletes, would appear to help meet NIH’s goals, the lack of funding for these studies may simply reflect NIH funding priorities. However, NIH did fund projects designed to examine the biomedical, but not athletic performance, correlates of DR, ACE, ACTN3, or MSTN during the sample period. Officially, NIH bases its funding decisions primarily on the scientific quality of proposals rather than targeting specific diseases but political pressure from disease advocacy organizations can have a large influence on NIH funding priorities (Best 2012). Therefore, the lack of NIH funding for studies specifically designed to focus on athletic performance is not surprising.

The NSF was created to “…to promote the progress of science; to advance the national health, prosperity, and welfare; to secure the national defense…” and funds basic biological research including that of physiological processes, development, and genetics (http://www.nsf.gov). Since 2000, NSF has funded projects designed to examine some of the correlates of MSTN in nonhumans, but not DR, ACE, or ACTN3 (http://www.nsf.gov/awardsearch). MSTN is of special interest to scientists because elucidating its biology may help provide therapies or cures for muscular dystrophy (Lee and McPherron 1999; Lee and McPherron 2001). This may help explain why 18 of the 89 (20%) of the authors on papers about the relationship between athletic performance and MSTN had USA addresses (Table 2).

### Pervasive “blank slate” thinking affects the publication patterns of USA based scientists

Finally, we argue that a pervasive environmentalist paradigm within the USA best described as “blank slate thinking” (Pinker 2002) may be ultimately responsible for the disproportionately small proportion of published research about the relationship between athletic performance and DR, ACE, ACTN3, or MSTN by USA based scientists. Blank slate thinking refers to the idea that the differences in performance among individuals are best explained by environmental differences among them during development (i.e., nurture) rather than by differences in their genetic endowments (i.e., nature) (Pinker 2002; Ridley 2003).

The influence of blank slate thinking on the practice of American science has varied since 1900 (Segerstråle 2000; Alcock 2001). Early in the 20^th^ Century many American scientists embraced the idea that genetic variation was largely responsible for individual and population variations in behavior (Segerstråle 2000). Indeed, many universities and states had departments or boards of eugenics (Kelves 1985). However, after World War II the intellectual focus shifted from biological to environmental explanations for human variation, including variations in social behavior (Segerstråle 2000). This shift occurred, at least partly, in response to the (a) atrocities of World War II resulting from Nazi philosophies of Aryan superiority and (b) 1952 UNESCO statement, “The Race Concept: Results of an Inquiry,” (http://unesdoc.unesco.org) that effectively banned biological research on human behavior (Segerstråle 2000; Selcer 2012). After the UNESCO statement, the “politically correct” view was that differences among individuals or groups were caused by differences in their social and cultural environments and had no biological bases. This perspective influenced the research programs of many scientists in the USA (Segerstråle 2000). Blank slate thinking retains a hold on many American academics and scientists (Segerstråle 2000) despite many of the recent theoretical and empirical advances in our understanding of how individual genetic variation and the environment synergistically influence human variation in the performance of a variety of different tasks (Pinker 2002; Ridley 2003; Plomin et al. 2008).

A growing body of scientific research directly challenges blank slate by revealing the important relationships between human genotypes and susceptibility to disease (e.g., Frank 2004; Tate and Goldstein 2004; Tishkoff and Kidd 2004). Indeed, physicians are coming to the realization that genotype is an important variable in considering disease diagnoses and treatment. Accordingly, NIH funds research on the relationship between genotype and disease susceptibility. The observation that NIH has funded several long several long-term studies of some physiological correlates of exercise (e.g., DREW (Morss et al. 2004), HERITAGE (Bouchard et al. 1995), INFLAME (Thompson et al. 2008), STRRIDE (Kraus et al. 2001)) that include both African-Americans and Americans of Caucasian descent as subjects is especially relevant to this study because it indicates that NIH is not adverse to funding projects that examine innate individual and population differences in physiology. This is interesting because the outcomes from studies that demonstrate individual, sex, and “racial” differences in physiology would appear to imply that these differences could result in innate differences in performance. Similarly, NSF has funded basic research on the relationship between genotypes and physiological phenotypes.

Nevertheless, the idea that innate differences among individuals play only a small role in producing differences in performance remains influential (e.g., Ericsson et al. 1993; Howe et al. 1998) despite the nearly global rejection by biologists of the idea that the phenotypic expression of morphological, physiological, and behavioral traits is determined by either genes or environment alone (Pinker 2002; Ridley 2003). Proponents of blank slate thinking provide a vivid example of how followers of the environmentalist paradigm explicitly reject modern theories and evidence about how traits are expressed. Sowell (1995) referred to the retention of incorrect theories in the face of contradictory data as the “irrelevance of evidence”.

In the end, athletic performance is a phenotype, and like all phenotypes results from the complex interactions between an individual’s genotype and its environment (Pinker 2002; Ridley 2003; Plomin et al. 2008). The lack of published scientific research originating from USA based scientists on the influence of innate variation on athletic performance is especially surprising because the hypothesis that athletic performance is not influenced by the innate differences among individuals in physical characteristics makes little biological sense.

Our findings beg the question; why don’t USA based scientists who conduct research on the relationships between genotypes and phenotypes and therefore, in general, appear to accept the idea that phenotypes result from the interaction between genes and environment, also publish scientific papers about the influences of innate variation on athletic performance? We argue that a history of slavery and its consequent explicit and implicit racism has made American scientists and USA governmental agencies like NIH and NSF reluctant about pursuing theories that posit innate variation as explanations for differences in athletic performance (Entine 2000, 2010; Smith and Hattery 2006; Smith 2007; Zirin 2008). For example, the legacy of the medical malfeasance of withholding treatment to syphilis-infected African-American men during the Tuskegee syphilis study has cast a long shadow on biomedical research in the USA (Reverby 2009).

A complete discussion of the discomfort Americans feel when confronting questions about how differences in athletic performance may be related to innate variation, especially the genetic variation among individuals of different geographic origin, is beyond the scope of this paper but is thoroughly covered by Entine (2000, 2010), Hoberman (1992, 1997) and Zirin (2008).

## Conclusions

Our scientometric analyses revealed that authors with USA addresses were underrepresented on scientific papers examining the relationships between athletic performance and innate variation in four characteristics (digit ratio and the genotypes for angiotensin converting enzyme, α-actinin-3, and myostatin) commonly associated with athletic performance. Regardless of the reasons for this pattern and what Entine (2010) calls “soft-censorship” in the USA of the scientific exploration of questions of how innate characteristics affect athletic performance, USA based scientists are failing to maintain pace with their colleagues elsewhere in the illumination of the factors that influence athletic performance because they fail to study possible innate correlates of performance. As a consequence, not only will the scientific study of sport by USA based scientists suffer, but so will the scientific study of the biological and environmental correlates of physical activity, fitness, and general health.

## Methods

### Web of Science search

We searched ISI Web of Science using the keywords “athlete(s)”, “athletic performance”, “sport(s)”, “digit ratio”, “angiotensin converting enzyme”, “ACE”, “α-actinin-3”, “ACTN3”, “myostatin,” and “MSTN” in various combinations to locate papers published on these topics between 1 January 2000 and 31 December 2012. We used the ISI Web of Science to collect scientometric data because of the utility of its citation analysis tools relative to some other databases (Falagas et al. 2008). Scientometry entails the quantitative measurement of scientific publications providing a way of quantitatively analyzing the relative contributions of individual researchers and research groups to the advancement of a field (Braun and Schubert 2007). Scientometric analyses can result in insights about the dynamics of advances of the field studied (Voracek and Loibi 2009).

We conducted two types of searches. First, we searched for papers that examined the relationship between athletic performance and each of the innate factors described above using the AND search function in Web of Science. The AND search function produced a list of publications that contained athletic performance and innate factor topics. Only papers (a) published in English in peer-reviewed journals, (b) focused on human biology, and (c) that examined the relationship between athletic performance and each of the innate factors described above were selected for analyses. Depending on their information content, we sequentially examined the title, abstract, then when necessary, the full text of papers to determine whether or not papers met our criteria for inclusion for review. It had to be clear from the title, abstract, or text of the paper that the innate factor in question was examined in relationship to athletic performance. We excluded from analyses papers about the relationship between an innate factor and general health or exercise performance not directly in the context of athletics. Working together, we screened the studies for inclusion for review based on the criteria described above and then jointly decided whether a paper was included or excluded from analyses. The papers that remained after we removed those that did not fit our inclusion criteria made up our experimental samples.

Second, we searched for papers about the innate factors not in the context of athletic performance using the NOT search function to produce our control sample. The NOT function produced lists of publications in which the innate factors were included as topics but sport(s), athlete(s), and athletic performance were not. We culled these lists of publications as described above. We used a random number generator to collect papers generated from the NOT “search” to equalize sample sizes between our experimental and control samples. For example, the search for papers about the relationship between athletic performance and ACE using the AND search function produced a list of 141 papers of which 74 met our inclusion criteria whereas the search for papers on ACE using the NOT search function produced a list of 22,031 papers. We then used a random number generator to collect 74 papers that fit our inclusion criteria in order to equalize the experimental and control group sample sizes (Table 1). We did this for each innate factor except ACTN3. The search using the NOT search function with ACTN3 produced a shorter list of papers than did the search using the AND search function. In this case, we searched the NOT “search” generated list for papers that satisfied our inclusion criteria (Table 1).

We collected the title, year published, journal where published, and each author’s name and national address from each paper that met our inclusion criteria. By convention, the address an author lists on a paper is typically their address when the work was performed. We collected the national addresses of all authors on all papers to compare the proportion of authors with USA addressees listed on papers that examined the relationships between athletic performance and our four innate factors with that listed on papers that examined the innate factors independent of athletic performance. To compare the proportions of papers that originated from either USA or non-USA based laboratories, we collected the national addresses of the first and corresponding author of each paper. To avoid statistical pseudoreplication we counted authors only once in each category (i.e., using either the AND or NOT search functions). Authors with addresses in England, Northern Ireland, Scotland, and Wales were grouped together in the United Kingdom.

To compare the patterns of publication by authors that listed USA addresses with those from other countries relative to the AND and NOT search function categories we performed three different comparisons. First, we examined the list of authors on papers found using the AND search function for the country, excluding the USA, that had the greatest number of different authors. We did this to compare the proportions of authors that listed their national address as that country on papers found using the AND and NOT search function for each of the four innate factors individually and for the factors pooled together. Second, we examined the list of authors on papers found using the NOT search function category for the country, excluding the USA, that had the most different authors. We did this to compare the proportions of authors that listed their national address as that country on papers found using the AND and NOT search function for each of the four innate factors individually and for the factors pooled together. Last, we compared the proportions of authors that listed the USA as their national address with those of authors that listed elsewhere as their national addresses found on papers using the AND and NOT search function with those of authors from the countries represented by the most authors found on papers using the AND and NOT search function for each of the four innate factors individually and for the factors pooled together. We performed these comparisons to detect whether the patterns of publication by authors with USA addresses were similar those of authors that listed their national addresses as elsewhere.

Finally, we restricted ourselves to Web of Science searches using the AND or NOT search functions as described above. Restricting ourselves to these types of searches ensured that our chances of finding suitable papers for review remained consistent each time we searched.

### NIH and NSF database searches

We used the keywords “athlete(s)”, “athletic performance”, “sport(s)”, “digit ratio”, “angiotensin converting enzyme”, “ACE”, “α-actinin-3”, “ACTN3”, “myostatin”, and “MSTN” in various combinations to search the US National Institutes of Health (http://www.nih.gov and projectreporter.nih.gov) and National Science Foundation (http://www.nsf.gov) databases to find solicitations for new grant proposals and new grant proposals funded between 2000–2012 that contained the keywords in their project titles or abstracts. NIH and NSF are the major USA governmental funding agencies for biomedical and basic biological research, respectively.

We used the keywords “race”, “racial differences”, “hypertension”, and “diabetes” in various combinations to search the NIH database to find grant proposals funded between 2000–2012 containing the keywords in their project titles or abstracts. We did these analyses to determine whether NIH funded biomedical research that examined racial correlates of hypertension and diabetes, both of which are common in the African-American population (Brancati et al. 2000; Adeyemo et al. 2009). If NIH funded research on these topics, then it would indicate that there are no organizational prohibitions of funding biomedical research examining the influences of innate individual and population differences in the development and presentation of disease. However, it is important to note that NIH funding for studies of diseases may be influenced by both their health consequences on a large segment of the population and, perhaps even more importantly, by political pressure exerted on governmental agencies by disease advocacy groups (Best 2012).

### Statistical analyses

We used Fisher exact tests to test the null hypotheses that (a) authors of papers about the relationships between athletic performance and DR, ACE, ACTN3, or MSTN were as likely to have USA addresses as non-USA addresses, (b) papers about the relationships between athletic performance and DR, ACE, ACTN3, or MSTN were as likely to have originated from USA as non-USA based laboratories as determined by the national addresses their first and corresponding authors, and (c) in context of being listed on papers about the relationships between athletic performance and DR, ACE, ACTN3 or MSTN, the patterns of publication by authors that listed USA addresses were the same as those of authors that listed elsewhere as their national addresses. The Fisher exact test has no statistical rival for these sorts of comparisons (Marascuilo and McSweeney 1977). We considered either a disproportionately small number of authors that listed USA address or papers originating from USA based laboratories as evidence that scientists that listed USA addresses did not pursue questions about the influence of these innate factors on athletic performance as frequently as did scientists that listed elsewhere as their national address. We used G*Power 3 software program, version 3.1.3 (http://www.gpower.hhu.de) to calculate the achieved statistical power, 1 – β = error probability (Cohen 1992), of statistically non-significant outcomes. All statistical tests were two-tailed. The probability level for statistical significance was set at α = 0.05. Unless otherwise noted, all values are reported as mean ± SD.

## Abbreviations

DR: Digit ratio
ACE: Angiotensin converting enzyme
ACTN3: α-actinin 3
MSTN: Myostatin.
